# Infra-low frequency neurofeedback in application to Tourette syndrome and other tic disorders: A clinical case series

**DOI:** 10.3389/fnhum.2022.891924

**Published:** 2022-08-16

**Authors:** Bodil Solberg, Erlend Solberg

**Affiliations:** Barn & Unges Potensial AS, Tvedestrand, Norway

**Keywords:** neurofeedback, Tourette disorder, tic disorder, child and adolescence psychiatry, brain training, neuropsychiatry, biofeedback

## Abstract

We describe our clinical experience in treating patients with Tourette syndrome and other tic disorders using infra-low frequency neurofeedback (ILF NF), often in conjunction with cognitive behavior therapy. Following a narrative description of our approach, we present outcome data for 100 successive cases. Many of the children and adolescents that we have treated since 2005 did not derive sufficient benefit from standard treatment for Tourette syndrome and other tic disorders. In our clinical experience, based on extensive before- and after- testing and symptom tracking, this patient group derived significant additive benefit from complementary neurofeedback treatment. The majority of trainees attained a higher level of functioning and were able to live up to their potential in a way they were not able to prior to neurofeedback treatment.

## Introduction

Our team consists of a Child and Adolescent Psychiatrist experienced in cognitive psychotherapy and a Neurofeedback Therapist. The Child and Adolescent Psychiatry Clinic is part of, and funded by the Norwegian national specialist healthcare system, and focuses on neuropsychiatric diagnostics, cognitive therapy and neurotherapy. Cases are seen strictly by referral, from hospitals or other treating professionals, by virtue of having failed conventional treatment.

Over the course of 17 years, we have become highly specialized in the treatment of Tourette syndrome and other tic disorders using neurofeedback and cognitive therapy. The standard treatment for Tourette and tic disorders has been medication, psychotherapy, academic support at school, and counseling of parents and school personnel. For those who derive insufficient benefit, suffer unacceptable side effects, or for other reasons cannot be medicated, there is a great need for additional treatment methods.

Typically, in this patient group about 30–40 neurofeedback sessions are required. Some patients benefit from 5 to 10 booster sessions later if problems reappear. The treatment is evaluated after 20 sessions, where the team and the patient, in cooperation with the parents, decide whether and how to continue the treatment.

## The neurofeedback-centric therapeutic approach

### Testing and diagnosis

Prior to Neurofeedback, each patient is tested and diagnosed using various standardized tools following the directives of the World Health Organization (WHO). The ICD 10 system is used for diagnostics. In addition to the diagnostic evaluations, we establish individual goals on a Goal Attainment Scale (GAS) that includes tic severity and we do symptom tracking before and after neurofeedback. If needed, especially if the patient is initially referred to us with an ADHD diagnosis, Conners CPT and ADHD Rating Scales are used.

### Understandable explanation of the complex process to create buy-in

It is extremely important for us to cooperate with patients/parents and other influential persons in the patient’s network. And it is necessary to explain the complex process of neurofeedback in a simplified and understandable way to get buy-in.

Core procedures and considerations in the Neurofeedback-Centric Therapeutic approach include: (1) testing and diagnosis, (2) classifying major symptom patterns, (3) determining optimal training frequencies, (4) choosing and revising cortical sites based upon the above information, and (5) careful, detailed clinical observation and ongoing symptom tracking. Training begins with one or two starting protocols, and additional sites may be added later. The combination of frequency optimization and the choice of sites individualizes the training for optimal results for each patient. The method is more fully described in the protocol guide, which is presently in its seventh edition (Othmer, 2019).

In 2-Channel ILF-NF training, the brain is subject to feedback reinforcement of the differential signal recorded from the two selected cortical sites. The differential training impinges on connectivity relationships. Site pairs are selected based on the initial testing and diagnostic particulars and are then iterated based on careful clinical observation and symptom tracking. Because of this, we explain to our patients and families the importance of careful observation and reporting of symptom status.

### Training at individually optimized frequencies

The training is conducted at individually optimized frequencies. The concept of “Optimal Response Frequency” (ORF) is explained by analogy to the fine-tuning of an analog radio channel. This involves adapting the training parameters so that the patient is relaxed, focused and in a calm and pleasant emotional state. Our goal is also for the patient to feel good when they are visiting us. During neurofeedback, and during the optimization procedure in particular, we observe both facial expressions and other body language, and attend to verbal cues, to try to gauge both state of mind and self-regulatory status of the patient.

### Major model #1 – dysregulation

Within the Dysregulation Model that underlies the present protocol schema, tics represent a signal from the brain that indicates a regulatory deficit. That is to say, tics lie in the functional domain, and ought to be susceptible to a functional remedy. The strong state dependence we observe with tics supports that basic assumption.

Dysregulation impacts many areas of the person’s life. We use the image of a car to explain core concepts arousal, excitability, and inhibition. Is acceleration too lively? Are the brakes too weak? Baseline arousal level is explained by analogy to the idling of a car. Excitability is explained in terms of how responsive the car is when the accelerator is pressed.

The patients in this group often have a lot of energy. We liken it to “the bubbles in a champagne bottle.” They are fighting to keep the cork in the bottle, but sometimes they fail. It is important to recognize that they are not trying to manipulate us; they are just doing things as well as they are able to.

We have two options: (a) to tighten the cork, which calls for left hemisphere training; or (b) to relieve the over-pressure and ease the bubbles, which calls for right hemisphere training. We usually start with “b” to calm the brain. The T4-P4 placement is a core starting placement for brain calming.

### Major model #2 – cerebral instabilities

Since the tics are episodic, they can also be understood in the frame of the instability model. More specifically, the fluctuation in incidence of tics scales with neuronal excitability. Within the scope of our protocol schema, both instabilities and neuronal excitability are targeted with bi-hemispheric placement at homotopic sites, principally T3-T4. Connectivity training with such bi-hemispheric placements aims at improved coordination between the two hemispheres for improved cerebral stability as well as a general calming of excitability.

The choice of T3-T4-training as the default starting placement is based on clinical experience for a variety of conditions, particularly members of the class of “cerebral instabilities.” Epilepsy is a case in point. Children with epilepsy as a comorbidity are also seen at our clinic, and for them T3-T4 is so central to their training toward stabilization that it has come to be referred to as the “lamotrigine site.” Most patients benefit from this training procedure, aiming at augmenting and consolidating what for these patients can be achieved with Antiepileptic medication.

## Judgment of progress through observation

Progress in training is judged mainly by observation and feedback from the patient and its network. When working with children, we recognize that body language is a big part of their way of expressing themselves. It is a challenge to learn how to interpret them by observing. We also query the parents but cannot always trust what the adults assume or believe. There are times when adults have their own motives for characterizing the behaviors of their children, and they may just misunderstand the child’s behavior.

### Symptom patterns

#### Stress and anxiety

Children and adolescents with TS are often very stressed and anxious. We aim to calm these symptoms using the T4-P4 placement, and later in training give them additional control by adding prefrontal training, predominantly on the right hemisphere, using the placement T4-Fp2. We often find that when these patients are referred to us, they are medicated with central stimulating medication (Methylphenidate, etc.) due to secondary attention problems. If the dose is too high, the noradrenergic effect often creates even more stress/anxiety and can either lead to hyper-excitability or, in worst case, emotional apathy.

#### Fears and oppositional behaviors

Many of the children and adolescents we treat have a lot of fears, and they exhibit oppositional behaviors. Training at T4-Fp2 usually calms emotional reactivity and increases the ability to self-regulate. When NF treatment is successful, and the patient calms down, we usually have to titrate the dosage to avoid over-medication issues. In addition to the desired calming effect, the patient often experiences enhanced ability to focus as a result of NF training. We expect them to be free of medication after 30/40 sessions, or in rare cases to require only a low dose of stabilizing medication (e.g., lamotrigine).

#### Lack of focus

Another problem that this patient group commonly faces is a lack of focus. And yet many of these patients cannot tolerate the left-side prefrontal training that one would ordinarily bring to bear on this issue. When this protocol is used, the training is often done at a lower frequency than usual to prevent over-activation. The same holds true with “chaotic thought” patterns.

#### Obsessive thoughts and hang-ups

Obsessive thoughts and hang-ups are commonly observed with TS patients. These behaviors are usually triggered by internal and external stressors and anxiety. Neurofeedback is very useful in calming down these symptoms, particularly in combination with cognitive behavior therapy (CBT). We use this approach instead of antidepressant medication. The cognitive therapy teaches them “how to think”’ regarding their obsessive thoughts. The neurofeedback effect here is twofold: during the course of neurofeedback, we often see that these patients forget their rituals and the things they earlier “had to do.” As they become less vulnerable during NF treatment, it enables them to participate in the exposure part of CBT.

#### Slow reading, perfectionism, and obsessiveness

Slow reading in this patient group can often be attributed to perfectionism and obsessiveness. It must be “right” before they go on; it has to be “perfect.” Teaching them about “good enough” is important when trying to correct this issue. Learning disabilities may also manifest as slowed processing speed. This can lead them to underperform in a school setting and on intelligence tests. In these patients we evaluate the T3-P3 placement before more specific sites are targeted according to symptom profile. NF training for “autistic traits” is focused on right-side placements, both posterior and anterior, to target arousal regulation and social skills, respectively. The same approach applies when treating PTSD and the consequences of early attachment problems.

#### Use of additional protocols

Thus far the discussion has covered only the standard starting protocols. If these are not sufficient, other strategies can be brought to bear. Some patients with inattention and high anxiety need both right-hemisphere calming and left-hemisphere activation. This can often be accomplished by combining standard beta-SMR band training in the left frontal region (T3-Fp1) with infra-low frequency neurofeedback (ILF NF) at T4-P4. Some sensitive children/youth respond better to interhemispheric training than to lateralized training.

## Results of formal assessments

### Goal Attainment Scale

The GAS is an individualized outcome measure that is used to ascertain whether the patient’s goals for the treatment have been met. Together with the parents we identify five important goals for the treatment and determine which specific objectives should be achieved in order to meet the goal criteria. The goals should be positive, concrete, and measurable. For example: manage to solve conflicts verbally (no physical violence); manage to start a homework assignment after age-appropriate reminder; be able to focus on reading a page in a book for 10 min; reduce tics by 50%.

Twenty-session evaluation: Are we going in the right direction with regards to the goals? What is left to be done? What do we prioritize in the further treatment? Any new problems? When do we stop treatment?

**FIGURE 1:**
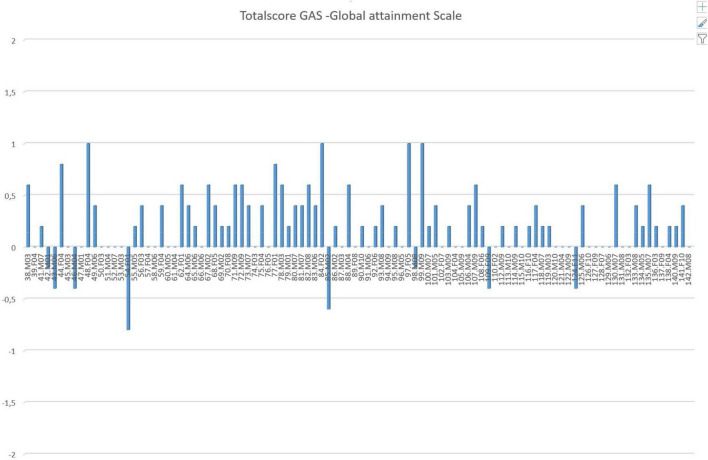
Goal Attainment Scale scores for the latest 100 patients with tic disorders. The treatment is considered successful if the GAS score is 0, matching the predicted outcome. At the end of training, scores are assigned to each of the five goals as follows: –2 = no effect, –1 = little effect, 0 = expected effect (goal of treatment), +1 = good effect, +2 = very good effect (issue is no longer a problem). These scores are summed over the five goals, and the net sum is then normalized. It is observed that over 90% met or exceeded the expected goals of the training. Subjects are arranged in consecutive order of time of treatment.

End evaluation: Evaluate daily functioning and goals together with patient/parents/school, pediatric/psychological services, and other important persons in the network. Results for the last 100 patients seen are shown in Figure 1.

### Symptom tracking

This tracking tool covers 155 symptoms classified into different areas, including the domains of sleep, attention, learning, sensory, behavioral, mood, physical, and pain.^1^ Before NF therapy we grade every symptom on a scale from 0 to 10, with 10 the most severe. The symptom tracking also gives us valuable information about the client’s personality profile.

**FIGURE 2:**
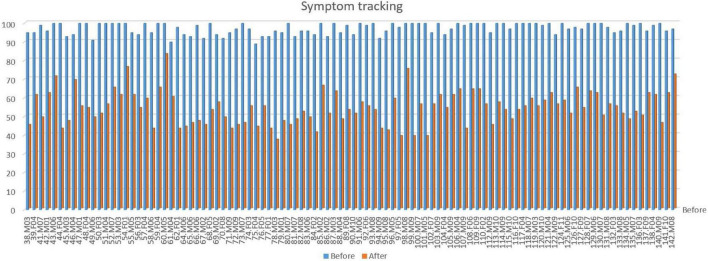
Symptom tracking scores for the latest 100 patients with tic disorders. The graph shows the total score of the 10 most severe problem areas before and after treatment, each scored on a 0–10 severity scale, for the cohort shown in Figure 1. Each pair of bars represents one patient. Average reduction in total score: 45% (female 44%, male 45%). No significant difference in improvement is seen between genders. Subjects are arranged in consecutive order of time of treatment.

**FIGURE 3:**
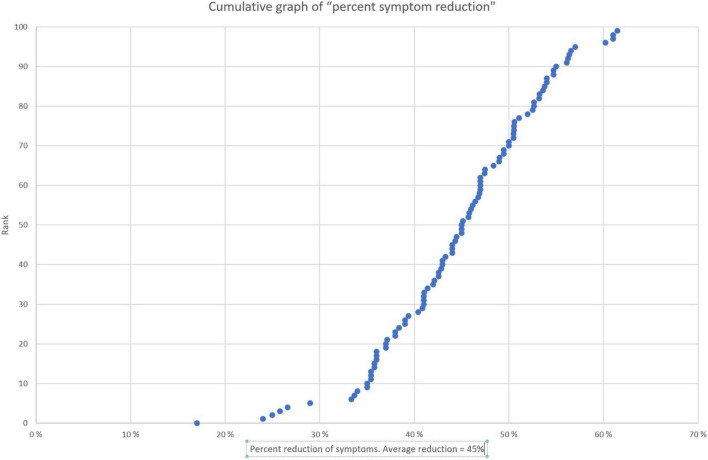
Cumulative graph of percent symptom reduction for the 10 most severe problem areas. The salient data shows the distribution in relative improvement. The plot is based on the same data as Figure 2, recalculated and displayed in rank order of increasing percent improvement. Each dot represents one individual. The median score was a 45% improvement and 90% improved by 35% or more.

Twenty-session and end evaluation: Together with parents, school, and others the 10 most severe problems are evaluated for change after treatment. These 10 are each given a value between 1 and 10 and are summarized to show a baseline before treatment. This is then repeated after NF treatment. In most cases we experience a significant reduction in severity with neurofeedback training. These results are shown in Figure 2 for the same patient cohort as Figure 1. Corresponding relative reduction in symptom severity is shown in Figure 3 in a cumulative distribution.

### Explanation of the data selection process

The last 100 patients that obtained a tic-related diagnosis between 2016 and 2020 are included in the evaluation data set, irrespective of level of severity. Data from 2005 to 2016 are not included due to changes in neurofeedback methods and instrumentation over that time frame.

All patients received neurofeedback as their main treatment, undergoing between 30 and 40 sessions. All were treated with the latest instrumentation (NeuroAmp II) and software (HD “High-Definition” ILF module) of Cygnet (BeeMedic, Germany). Visual and auditory feedback was provided by way of video games (Somatic Vision^2^). In addition, some were treated with Cognitive Behavioral Therapy, individual and family support and other measures if needed.

Each client has a code that consists of a consecutive number. M for male, F for female, and year of birth. For example: 178.M10 or 179.F11. Age range was between 8 and 18. The average age was 12 years. In total, 65 males and 35 females are included in the evaluation data.

## The long-term effects of neurofeedback

Since starting neurofeedback 2005, we have not performed any formal follow-up studies of the long-term effects of the treatment. However, we do have reliable informal indicators of the long-term effect from neurofeedback. Operating in a small community in Norway, we often get positive feedback from parents, health professionals, schools, clients, and parents.

Another indicator of long-term effect is the number of former clients who return to us after partially falling back into problematic behaviors and symptoms. Although surprisingly rare (approximately 1/10) we have observed that this can happen with complex, sensitive and volatile nervous systems. Symptoms can also worsen when the client enters adolescence, and the brain is under heavy “reconstruction.” Other major events and traumas in their life can also trigger a symptom relapse. In these cases, we will usually invite the client for several booster sessions, to remind the brain to get back on track. Our experience is that 5–10 sessions are usually sufficient.

Social situations, family dynamics, poor parental skills, and other negative environmental influences are other important factors that are crucial for a child’s development and can disrupt the long-term effect of neurofeedback. Additional resources then need to be recruited as well such as parental support and/or other social services.

Most clients will have gained sufficient self-regulation, robustness, and resilience so that they will function better in their normal environment and allow for them to mature in a more positive direction. Many will still need some extra help at school, in order to function optimally, but usually less than before.

## Conclusion

Most children/youths referred to us since 2005 had not derived sufficient benefit from standard treatment for Tourette syndrome and other tic disorders. In our clinical experience, based on extensive before- and after- testing and symptom tracking, this patient group derived significant benefit from complementary treatment with ILF neurofeedback. The substantial majority of trainees attains a higher level of functioning, allowing them to live up to their potential and to mature in a way they were not able to prior to neurofeedback treatment.

## Data availability statement

The raw data supporting the conclusions of this article will be made available by the authors, without undue reservation.

## Author contributions

BS wrote the manuscript based on patient data. ES created the database and performed the statistical analysis. Both authors contributed to manuscript revision, read, and approved the submitted version.

## Footnotes

1
www.eegexpert.net

2
http://somaticvision.com
